# Supplementary material for: Computational Understanding of Delithiation, Overlithiation, and Transport Properties in Disordered Cubic Rock-Salt Type Li2TiS3
Source: Nanomaterials (Basel). 2023 Nov 24;13(23):3013. doi: 10.3390/nano13233013 (PMC10707874; doi:10.3390/nano13233013)
Supplement: Supplementary file 1 [file nanomaterials-13-03013-s001.zip › nanomaterials-2699332-supplementary.pdf]

Computational Understanding of Delithiation, Overlithiation, and Transport Properties in Disordered Cubic Rock-Salt Type Li_2TiS_3. Supplementary Materials

Riccardo Rocca [1,2*], Mauro Francesco Sgroi [1], Maddalena D'Amore [1], Nello Li Pira [2] and Anna Maria Ferrari [1,*]

[1] Department of Chemistry and NIS, University of Turin, 10125 Torino, Italy; maurofrancesco.sgroi@unito.it (M.F.S.); maddalena.damore@unito.it (M.D.)
[2] Centro Ricerche FIAT S.C.p.A, 10043 Orbassano, Italy
* Correspondence: riccardo.rocca@unito.it or riccardo.rocca@external.crf.it (R.R.); anna.ferrari@unito.it (A.M.F.)

Table S1. Number of possible configurations based on the number of Lithium atoms removed from the structure. Calculated with CRYSTAL code.

Delithiation (%)	Li atoms removed	Configurations
100	18	1
94.44	17	18
88.89	16	153
83.33	15	816
77.78	14	3060
72.22	13	8568
66.67	12	18564
61.11	11	31824
55.56	10	43758
50	9	48620
44.44	8	43758
38.89	7	31824
33.33	6	18564
27.78	5	8568
22.22	4	3060
16.67	3	816
11.11	2	153
5.56	1	18
0	0	1

Table S2. Relative stability of three configurations each delithiation state. The most stable structure is considered for the energy-delta calculation.

x	Relative stability (eV)	x	Relative stability (eV)
**2**	0.000	**9**	0.000
	0.080		1.111
	0.142		3.538
**3**	0.000	**10**	0.000
	1.274		0.078
	1.561		0.210
**4**	0.000	**11**	0.000
	0.135		2.791
	1.229		3.630
**5**	0.000	**12**	0.000
	1.940		0.215
	2.285		1.594
**6**	0.000	**13**	0.000
	0.173		1.181
	0.883		2.653
**7**	0.000	**14**	0.000
	1.100		0.119

	1.503		0.154
**8**	0.000	**15**	0.000
	0.218		1.803
	0.939		3.102

Table S3. Reaction energy for the most stable structure for each delithiation state, x is the number of Li atoms removed. Also reported the required energy for a single lithium extraction.

x	$\Delta E_{Reaction}$ (eV)	$\Delta E_{Li\ removal}$ (eV)
**1**	4.18	4.18
**2**	6.31	2.14
**3**	10.22	3.90
**4**	12.63	2.41
**5**	16.29	3.66
**6**	18.30	2.01
**7**	22.18	3.00
**8**	24.35	2.17
**9**	27.21	2.86
**10**	30.33	3.12
**11**	32.75	2.42
**12**	34.87	2.12
**13**	37.80	2.93
**14**	41.34	3.54
**15**	43.93	2.60

Table S4. Band gap evolution as a function of delithiation for structures with even vacancies.

x	Band Gap (eV)
0	2.46
2	2.35
4	2.39
6	2.31
8	2.23
10	2.13
12	2.27
14	2.15

Table S5. State of charge and open circuit voltage for the LTS system.

SOC (%)	OCV (V)
0	
5.88	1.42
11.76	1.51
17.64	2.43
23.52	2.33

29.41	2.64
35.29	2.61
41.17	2.83
47.05	2.77
52.94	2.80
58.82	2.73
64.70	2.74
70.58	2.78
76.47	2.75
82.35	2.70
88.23	2.72
94.11	2.77
100	2.76

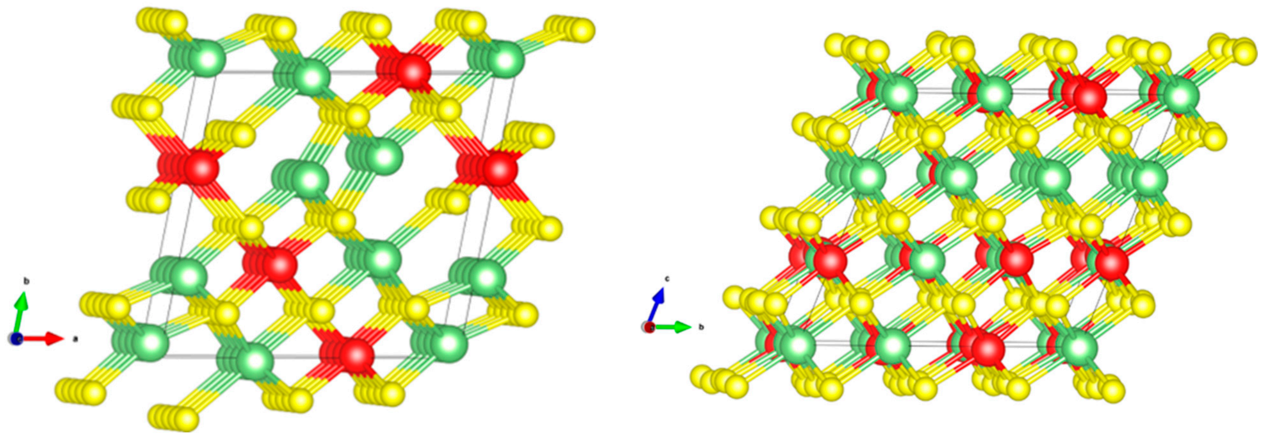

Figure S1. An example of ordered (left) and dispersed (right) structures.

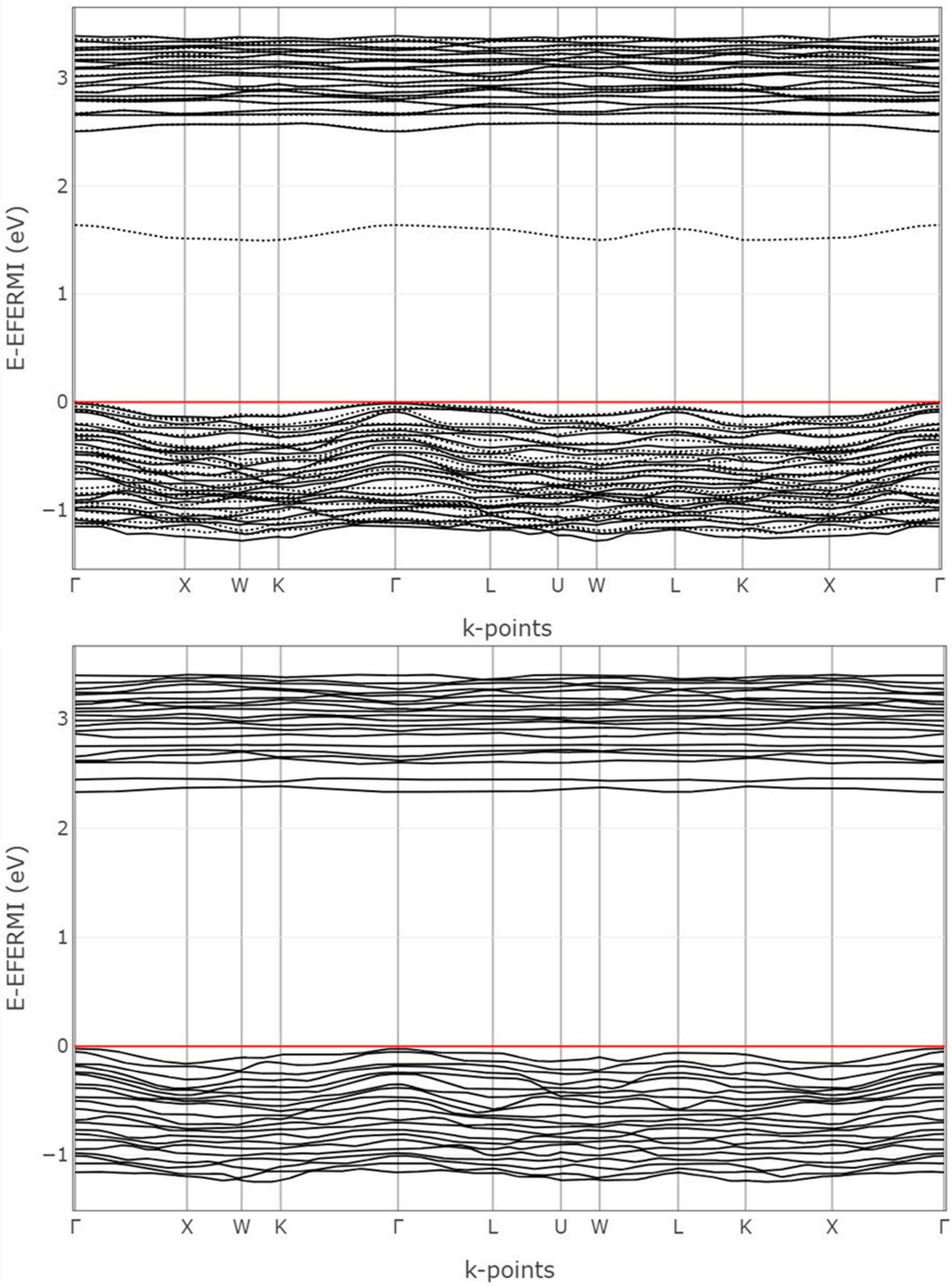

Figure S2. Band structure for mono-delithiated (top) and double-delithiated (bottom) structures.

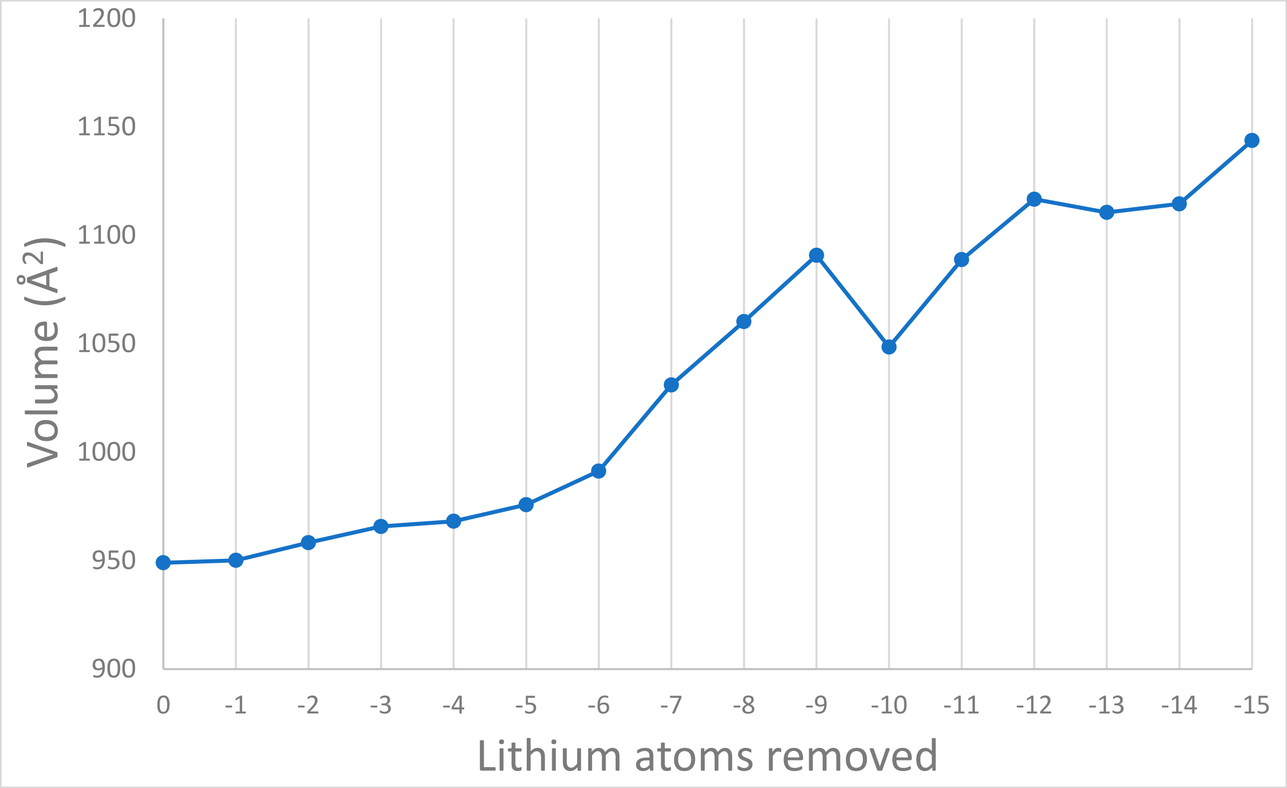

Figure S3. Volume variation during delithiation expressed in Å².

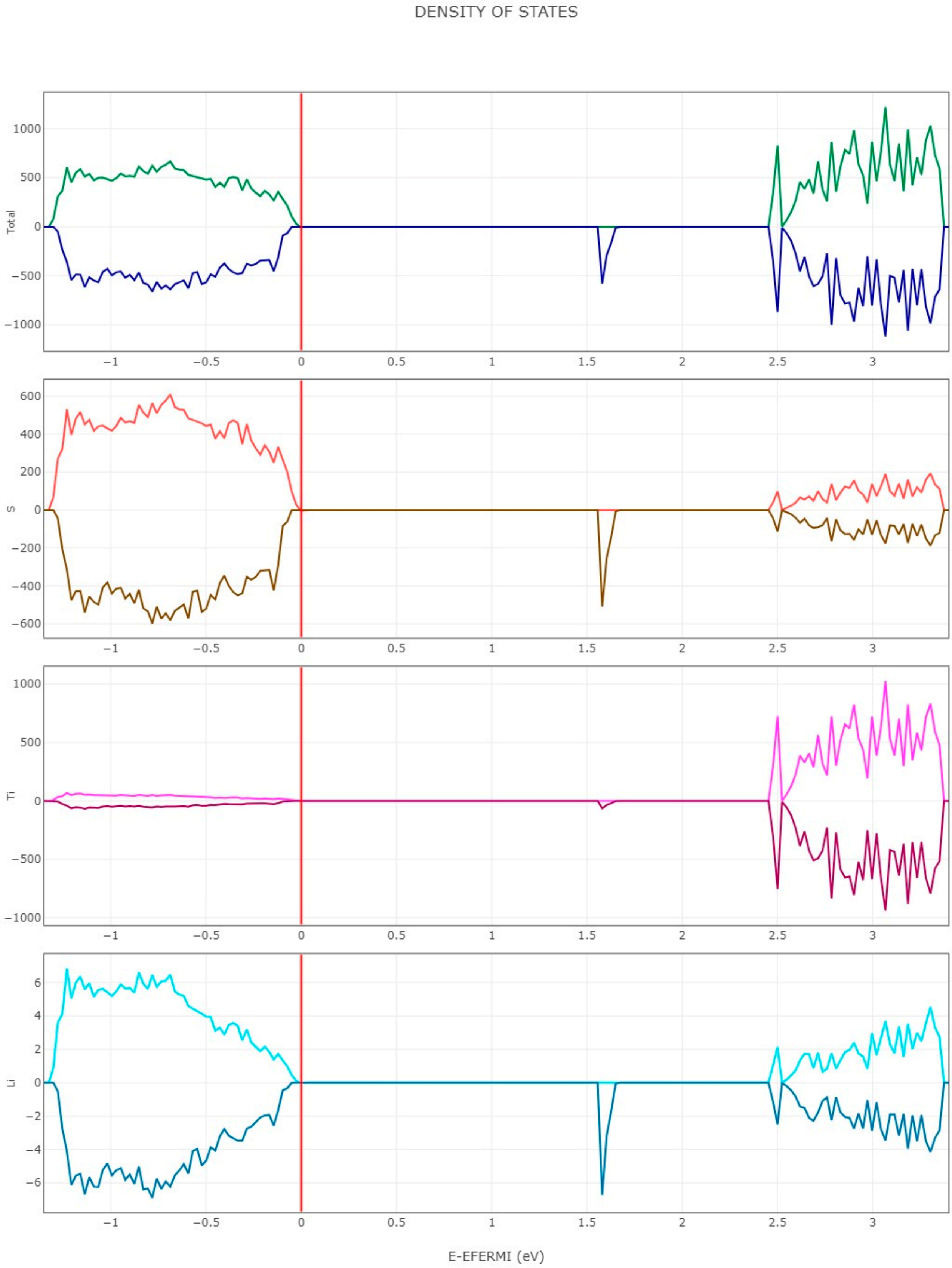

Figure S4. Density of states for mono-delithiated structure. Total DOS (top) and projections on atoms, from top to bottom: S, Ti, Li.
